# A scoring system to detect fixed airflow limitation in smokers from simple easy-to-use parameters

**DOI:** 10.1038/s41598-018-31198-8

**Published:** 2018-09-06

**Authors:** Esther Román-Conejos, Antonio Palazón-Bru, David Manuel Folgado-de la Rosa, Manuel Sánchez-Molla, María Mercedes Rizo-Baeza, Vicente Francisco Gil-Guillén, Ernesto Cortés-Castell

**Affiliations:** 1 Raval Health Centre, Generalitat Valenciana, Elche, Alicante Spain; 2 Department of Clinical Medicine, Miguel Hernández University, San Juan de Alicante, Alicante, Spain; 3 Department of Medical Management, University General Hospital of Elche, Elche, Alicante Spain; 4 Department of Nursing, University of Alicante, San Vicente del Raspeig, Alicante, Spain; 5 Department of Pharmacology, Pediatrics and Organic Chemistry, Miguel Hernández University, San Juan de Alicante, Alicante, Spain

## Abstract

No validated screening method currently exists for Chronic Obstructive Pulmonary Disease (COPD) in smokers. Therefore, we constructed a predictive model with simple parameters that can be applied for COPD screening to detect fixed airflow limitation. This observational cross-sectional study included a random sample of 222 smokers with no previous diagnosis of COPD undertaken in a Spanish region in 2014–2016. The main variable was fixed airflow limitation by spirometry. The secondary variables (COPD factors) were: age, gender, smoking (pack-years and Fagerström test), body mass index, educational level, respiratory symptoms and exacerbations. A points system was developed to predict fixed airflow limitation based on secondary variables. The model was validated internally through bootstrapping, determining discrimination and calibration. The system was then integrated into a mobile application for Android. Fifty-seven patients (25.7%) presented fixed airflow limitation. The points system included as predictors: age, pack-years, Fagerström test and presence of respiratory symptoms. Internal validation of the system was very satisfactory, both in discrimination and calibration. In conclusion, a points system has been constructed to predict fixed airflow limitation in smokers with no previous COPD. This system can be integrated as a screening tool, though it should be externally validated in other geographical regions.

## Introduction

Chronic obstructive pulmonary disease (COPD) is a major public health problem, making it the fourth leading cause of death worldwide^1^. Despite its associated morbidity and mortality and the considerable expenditure of resources generated by COPD, it is underdiagnosed worldwide^2–4^.

The main risk factor for COPD is smoking^5^. Other factors include age, male gender and respiratory symptoms^6^. COPD has also been associated with certain chronic comorbidities such as hypertension, cardiovascular problems, and diabetes^7,8^.

The most commonly accepted method to confirm COPD is forced spirometry, which is considered positive with a post-bronchodilator forced expiratory volume in the first second (FEV_1_)/forced vital capacity (FVC) ratio < 0.7. This is a simple, reproducible technique with low individual cost^9,10^. Although it is indicated in patients with respiratory symptoms, in normal clinical practice it is underutilized^11^, thereby explaining part of the underdiagnosis. Thus, forced spirometry does not appear to be a good screening technique that can be applied in all smokers regardless of their symptoms because, in most cases, these symptoms become evident when the disease is already established or when they already affect quality of life, which is associated with a greater severity of the disease^12,13^.

As there is no established early screening program, alternatives for the early detection of COPD have been proposed. These may prove useful prior to confirmation by spirometry to reduce the high prevalence of underdiagnosis. There are only three models to predict COPD in patients with a high risk of developing the disorder but with no prior history of lung disease (Table 1). Although these models are easy to use in general clinical practice as they employ variables that are easy to obtain and the risk of COPD is calculated with a scoring system, they nevertheless suffer from important limitations concerning their development (estimation) and their internal validation. Concerning the former, two of the models present overfitting (events-per-variable < 10), none of the three analysed the functional form of the continuous predictors and the treatment of missing data is not given; in addition to which only one model selected the predictors based on the overall goodness of fit of the model. Concerning the validation, this was not done by bootstrapping in any of the models and the calibration was not done with smooth curves to estimate the observed probability of COPD^14–16^. Accordingly, no real model to predict COPD is available for use as a screening tool developed with the best and recommended statistical technique.

**Table 1:** Analysis of the published studies that developed a predictive model to diagnose chronic obstructive pulmonary disease in risk populations (screening).

Reference	Population	Variables	Clinical use	EPV ≥ 10	Continuous variables	Missing data	Model building strategy	Discrimination
López-Varela *et al*.^38^	COPD risk	Gender, age, pack-years, dyspnoea, chronic phlegm and cough, and previous spirometry	Scoring system	Yes	Categorization	Not mentioned	Used all their co-variables	AUC, sensitivity and specificity, no bootstrapping
Llordés *et al*.^39^	Smokers	Age, gender, BMI, pack-years, profession of risk, expectoration, dyspnoea, cold complications, dyspnoea treatment and cardiovascular disease	Scoring system	No	Categorization	Not mentioned	Selection based on bivariate analysis	AUC, sensitivity and specificity, no bootstrapping
Price *et al*.^40,41^	COPD risk	Age, BMI, pack-years, cough due to the weather, phlegm, breathlessness and allergies	Scoring system	No	Categorization	Not mentioned	Stepwise selection based on bivariate analyses, factor analysis and AIC	AUC, sensitivity and specificity, no bootstrapping

Abbreviations: A.I.C., Akaike’s Information Criterion; A.U.C., area under the receiver operating characteristic curve; B.M.I., body mass index; C.O.P.D., chronic obstructive pulmonary disease; E.P.V., events-per-variable; C.O.P.D. risk is defined as being current or former smoker. Note that it is entirely possible that the mentioned limitations could be overcome by a subsequent successful external validation.

As a response to the need to implement a screening method that can differentiate smokers with a higher risk of fixed airflow limitation prior to onset of established symptoms, the objective of this study was the elaboration and internal validation (bootstrapping) of a prediction model for fixed airflow limitation, using the recommended statistical methodology^14–16^, with simple parameters that can be implemented in routine clinical practice in a systematic way, without incurring an increase in care time. In addition, to facilitate the implementation of this model, it will be integrated into a mobile phone application in the Android operating system (*COPD predictor* in Google Play), which could be used even by patients. With all this, we will have a screening tool that, provided it is externally validated in other populations, could be integrated into the protocols and clinical guidelines for COPD, prior to the definitive diagnosis by forced spirometry and respiratory symptoms. Whilst COPD screening is not currently recommended in smokers, this is because the techniques to do so are time consuming and expensive. Therefore, in this study we present an alternative that overcomes these limitations and leaves spirometry for confirmation in high-risk cases.

## Method

### Study Population

Smokers aged 40–75 years with no previous diagnosis of COPD whose primary health care is delivered at the Raval Health Centre in Elche (Alicante). This centre covers a population of 20,284 adults and is a primary care centre of the Spanish Public Health System which provides universal and free coverage.

### Study design and participants

This cross-sectional observational study was carried out during 2014–2016 with the objective of determining the underdiagnosis of COPD resulting from the underuse of forced spirometry and with this to construct a predictive model. We consulted the outpatient database of this centre and excluded those patients who did not meet the following inclusion criteria: smokers, age 40–75 years, and with no active diagnosis of COPD in their medical record. From the list obtained in this consultation, a group of patients was selected by random sampling (random number table) and contacted by telephone, asking them over the phone for their informed consent to participate in the study. The patients who agreed to participate were asked to perform forced spirometry, fill out questionnaires on COPD risk factors, and sign the written informed consent. Just under 10% of those selected refused to participate (generally due to incompatible work schedules, n = 24, 9.8%). These patients were passed over and the next person in the random table was selected.

### Variables and measurements

The primary variable was fixed airflow limitation performed with a validated Datospir 110 A spirometer, according to the regulations of the main Spanish medical societies and the criteria established in the GOLD guide (post-bronchodilator FEV_1_/FVC ratio < 0.7)^12,17^.

Secondary variables were collected according to the risk factors associated with the diagnosis of COPD: older age, male gender, number of cigarettes smoked and for how long, low body mass index (BMI), low educational level, presence of respiratory symptoms and exacerbations during the last year^4,8,18–20^. In the interview details were obtained about: gender; age; educational level (0 = primary, 1 = secondary-intermediate degree, 2 = higher studies-University); smoking history in pack-years, calculated as the number of cigarettes smoked per day multiplied by the number of years smoking divided by 20; Fagerström test, considering three levels of dependence: mild (0–3 points), moderate (4–6 points) and severe (7–10 points)^21^; presence of symptoms or respiratory problems (aphonia, cough, catarrh, bronchitis, etc.) requiring medical consultation during the last year and level of exacerbation, described as a sustained deterioration in the patient’s baseline clinical condition, beyond the usual daily variability, which appears acutely, and is accompanied by increased dyspnoea and expectoration and a change in the appearance of sputum, or any combination of these three symptoms, requiring a therapeutic change and classified as mild (no episodes of bronchitis in the last year), moderate (episodes treated by primary care physician) and severe (treated in the emergency area and/or hospitalisation)^22^.

### Sample size calculation

Since the objective of this study was to construct a predictive model of a binary event through a binary logistic regression model, the sample size had to verify that the ratio between the number of events and the number of predictors of the model was greater than or equal to 10^23^.

### Statistical analysis

Qualitative variables were described using absolute and relative frequencies, whilst quantitative variables were expressed through means and standard deviations. Our variables had no missing data. Associations between the primary variable and the secondary variables were assessed using the X^2^ test and the *t*-test. A logistic regression model was constructed with a maximum number of five predictors (57 patients with fixed airflow limitation). Taking into account that we had a total of 8 predictors [considering educational level and the Fagerström test as linear predictors, as they did not show a quadratic trend (Wald test)], we checked all possible combinations of 1 to 5 predictors, selecting the one with the highest discriminating capacity, that is, the one that gave a maximum area under the receiver operating characteristic curve (AUC). Thus, the AUC was calculated in a total of 218 combinations. The optimum combination was adapted to a points system using the Framingham study methodology^24^ which, through a weighting of the model coefficients and a categorization of the predictors, associates a score to each variable and the sum of these scores gives an event probability. Once the points system was developed, it was internally validated through bootstrapping (1000 random samples), since this is the most recommended technique^25^. Discrimination was determined in each of the 1000 samples (the points system can differentiate fixed airflow limitation) and calibration (verifying that the prediction of the model corresponds to reality). Discrimination is addressed by calculating the AUC, whilst calibration is evaluated through the construction of smooth curves (linear splines) with the Hosmer-Lemeshow test, which is appropriate for the recommended level of calibration (moderate)^15^. All analyses were performed with a type I error of 5%, and for each relevant parameter its associated confidence interval (CI) was calculated. Statistical packages used were SPSS Statistics 24 and R 2.13.2.

### Ethical approval

This project was approved by the Clinical Trials Ethics Committee of the Department of Health of the Generalitat Valenciana (General University Hospital of Elche, Alicante) at its meeting on 20 March 2014, in accordance with the International Guidelines for Ethical Review of Epidemiological Studies and the recommendations of the Spanish Society of Epidemiology on the review of the ethical aspects of epidemiological research studies, respecting the rules set forth in Law 15/1999 on Personal Data Protection in research studies. The committee approved the age range of the patients in the study (40–75 years), as the highest prevalence of COPD occurs at these ages.

### How This Fits In

The diagnosis of COPD is customarily made using forced spirometry and respiratory symptoms, but this does not appear to be a good screening technique that can be extended to all smokers. We elaborated and internally validated a prediction model for fixed airflow limitation with simple parameters that can be implemented in routine clinical practice in a systematic way. Care time would not be increased, since the model will be implemented in a mobile app for Android. With all this, we will have a screening tool that could be integrated, after external validation, in the protocols and clinical guidelines for COPD.

## Results

**Figure 1:**
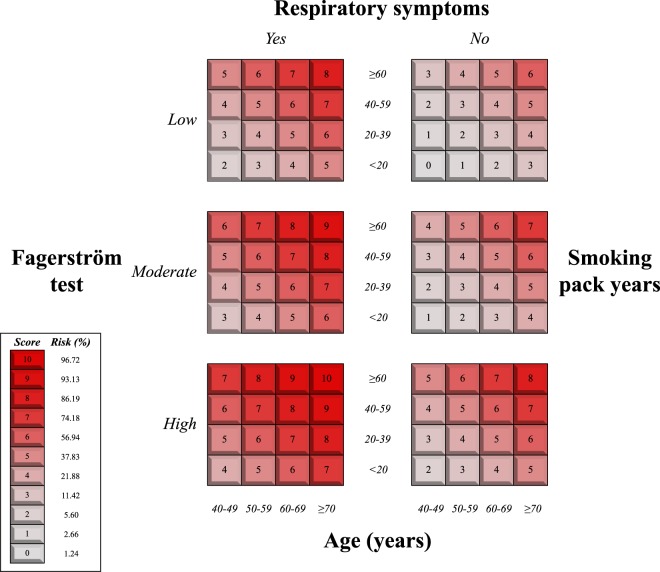
Scoring system to predict the diagnosis of fixed airflow limitation in smokers. The box on the left shows the overall scores with their associated risk of fixed airflow limitation.

**Figure 2:**
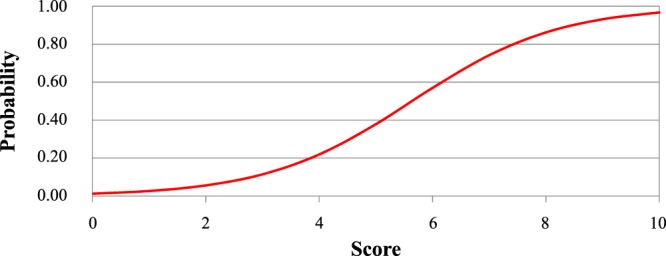
Predicted probability of fixed airflow limitation using our scoring system.

Spirometry was performed in a total of 222 patients, of which 119 (53.6%) were men; 57 (25.7%) patients had fixed airflow limitation. Table 2 shows the values of the variables analysed; also shown are the results of the bivariate analysis which revealed male gender, age, educational level, nicotine dependence (Fagerström), cigarette consumption, presence of respiratory symptoms and exacerbations to be significant risk factors (p < 0.05). The same table shows the coefficients of the multivariate model with the optimum combination to predict fixed airflow limitation. We highlight that this combination included: age, educational level, Fagerström test, pack-years smoked and presence of respiratory symptoms. Figure 1 illustrates the adaptation of the model to a points system (educational level was not present when we adapted the model to a scoring system). Figure 2 depicts the logistic tendency for the probability of fixed airflow limitation as the overall score increases.

**Table 2:** Predictive model for fixed airflow limitation in smokers with no previous diagnosis of chronic obstructive pulmonary disease in primary health care.

Variable	Total n = 222 n(%)/x ± s	Fixed airflow limitation n = 57(25.7%) n(%)/x ± s	p-value	Adj. OR (95% CI)	p-value
Male gender	119(53.6)	37(31.1)	0.047	N/M	N/M
Age (years)	56.8 ± 10.4	63.8 ± 9.2	<0.001	1.08(1.03–1.14)	0.001
Educational level*:					
Primary Secondary University	78(35.1) 87(39.2) 57(25.7)	29(37.2) 19(21.8) 9(15.8)	0.011	1.03(0.61–1.75)	0.902
Fagerström test (dependence)*:					
Low Moderate High	90(40.5) 113(50.9) 19(8.6)	12(13.3) 33(29.2) 12(63.2)	<0.001	2.06(1.07–3.99)	0.032
Smoking pack-years	28.8 ± 13.4	38.6 ± 11.8	<0.001	1.04(1.01–1.07)	0.021
Respiratory symptoms	88(39.6)	41(46.6)	<0.001	4.36(2.01–9.46)	<0.001
Exacerbation*:					
Mild Moderate Severe	147(66.2) 67(30.2) 8(3.6)	22(15.0) 28(41.8) 7(87.5)	<0.001	N/M	N/M
BMI (kg/m^2^)	26.6 ± 4.8	27.1 ± 5.8	0.387	N/M	N/M

Abbreviations: Adj. O.R., adjusted odds ratio; B.M.I., body mass index; C.I., confidence interval; n(%), absolute frequency (relative frequency) N/M, not in the model; x ± s, mean ± standard deviation. *Analysed in the multivariate model as a quantitative variable. Goodness-of-fit of the multivariate model (likelihood ratio test): χ^2^ = 11.5, p < 0.001.

**Figure 3:**
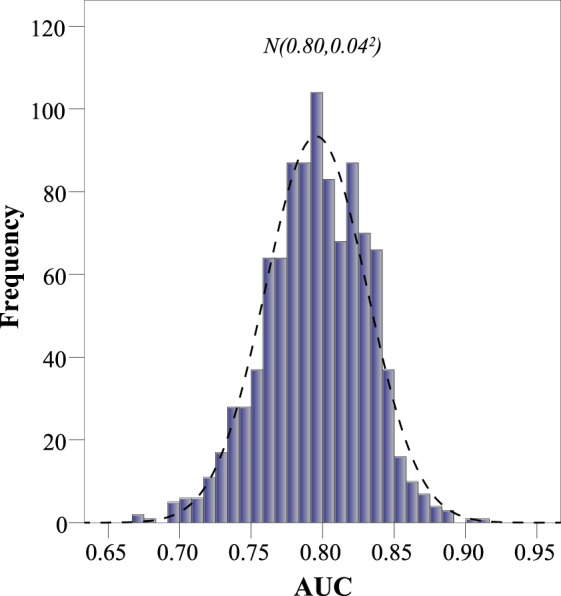
Area under the Receiver Operating Characteristic curve distribution for the validation of our scoring system using the bootstrap method. Abbreviations: AUC, area under the Receiver Operating Characteristic curve; CI, confidence interval.

**Figure 4:**
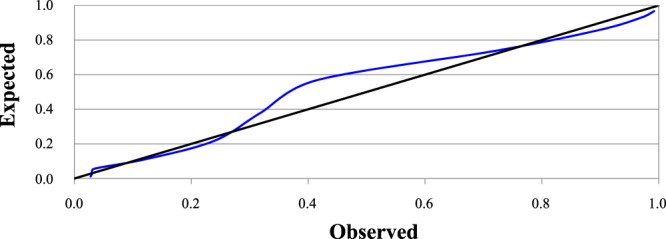
Smooth calibration plots for the validation of our scoring system using the bootstrap method. The black line represents perfect calibration and the grey line indicates the results of our calibration. The error (observed-expected in %) in each score was (ordered from 0 to 10 points; in parentheses we indicate the proportion of patients with each score): 1.53 (7.7%), 0.34 (5.9%), −2.30 (18.9%), 1.08 (17.6%), 2.11 (14.0%), −6.05 (12.6%), −15.36 (10.8%), −1.53 (5.0%), 4.04 (5.4%), 3.95 (1.8%) and 2.48% (0.5%).

The internal validation of the points system using the bootstrap technique was very satisfactory. First, in Fig. 3 we can see that the discrimination is adequate, as the central value of the AUC distribution is 0.80. Subsequently, Fig. 4 shows that the observed versus expected probabilities. Note that most of the scores had small errors, except when the patient had 6 points (15.36%), but these patients had a proportion of 10.8% in the sample and overall this was appropriate (Hosmer-Lemeshow test: p = 0.492). Consequently, the points system has been internally validated with satisfactory calibration and discrimination.

## Discussion

### Summary

This study developed a simple prediction tool to assess fixed airflow limitation in smokers with no previous diagnosis of COPD. The model is simple to use because the variables are easy to measure, risk is assessed by a points system and its implementation in a mobile application makes calculations even easier. The model has been internally validated in our setting through the most recommended statistical methods. Underdiagnosis of COPD in our population was 25.7% (fixed airflow limitation), with the following associated risk factors: male gender, older age, lower educational level, higher level of nicotine dependence (Fagerström), higher cigarette consumption, presence of respiratory symptoms and high level of exacerbations.

### Strengths and limitations

The main strength of our study is the development and internal validation of a prediction model for COPD screening in patients prior to diagnostic confirmation by spirometry and respiratory symptoms. Although others have developed a prediction model, their models have several limitations for use in clinical practice (Table 1). We also highlight the statistical methodology used since we chose the combination of explanatory variables of the multivariate model with the highest discriminating capacity. Additionally, we used the most recommended techniques for the validation of a predictive model of a binary event^14–16,25^.

Selection bias was minimized by randomly choosing the sample from among all smokers in the corresponding health area, with very low exclusion for not wanting to participate (about 10%). To minimize information bias, the tests were conducted by the principal investigator using validated questionnaires and instruments. To avoid possible confounding bias, we applied well-calibrated multivariate models with high discriminating capacity. Our main limitations were sample size (limiting the number of predictors) and lack of external validation (future line of research).

We could have used the lower limit criterion instead of setting the FEV_1_/FVC ratio threshold at 0.7. However, it has been seen in populations similar to ours that establishing the diagnosis of COPD with the lower limit could exclude a high number of patients with significant clinical impact and high consumption of healthcare resources^26^. We have also used a total of 8 explanatory variables to predict fixed airflow limitation, with a greater number of factors contributing to this problem. Nevertheless, the mathematical model had very good discrimination and good calibration.

Another point to take into account is that we did not have a sufficient sample size to externally validate the points system constructed. For this, a completely different sample should be available with at least 100 patients with fixed airflow limitation^16^. Our team is collecting a new sample for this purpose, and this type of study should also be carried out in other geographical areas to determine whether the predictive model is satisfactory for detecting fixed airflow limitation.

We would like to note that our points system is only applicable to patients without COPD who are current smokers and between the ages of 40 and 75 years. Patients with COPD who have no history of smoking have also been excluded, as they already have the disease and there is no point in screening. The exclusion of ex-smokers and elderly people could lead to an increase in the prevalence of fixed airflow limitation, and it should be verified in another study whether the model is applicable to these people, since the variables age, Fagerström test, and pack-years can be evaluated in these excluded patients. If similar results are obtained, the target population of our predictive model could be expanded.

### Comparison with existing literature

The technique used to confirm the presence of COPD is still spirometry and respiratory symptoms^12^, but its use is limited and the effectiveness of extending it to the entire population is unknown^11^. Consequently, several authors have proposed the possibility of first using questionnaires aimed at detecting and classifying patients at high risk of COPD, in conjunction with the intermediate use of a pocket spirometer, and followed by confirmation with conventional spirometry in cases with low FEV_1_/FEV_6_ figures^11,27^.

The older predictive models had major statistical limitations (Table 1). We used the recommended guidelines for developing a predictive model^14–16^ in order to develop a simple algorithm based on the data obtained in our study indicating the population at risk of COPD and that could go undetected. The tool can be used quickly in daily clinical practice by the primary care physician, or even with the development of a mobile application (app) that facilitates self-diagnosis by the patient (fixed airflow limitation), leading directly to the need for spirometry and assessment of respiratory symptoms to confirm the presence or absence of COPD and thus preventing the high levels of underdiagnosis. Consequently, this tool can be very useful, since the estimated worldwide prevalence of COPD is 1% in the general population and 8–10% in those aged over 40 years^28^, and between 2.1% to 26.1% in Europe depending on country, method and population^29^.

Regarding risk factors associated with underdiagnosis, age and smoking are already recognized as the main risk factors for underdiagnosis^3,12,20,30–34^. Other studies refer to a higher risk of underdiagnosed COPD in cases of low educational level^3^, low socioeconomic status^20^, previous respiratory symptoms or high BMI (≥30 kg/m^2^)^2^, whilst others associate it with low BMI^11^. Underdiagnosis has also been associated with increased comorbidity, especially cardiovascular^5,19,32,35,36^. Therefore, we can confirm correspondence with the data found in our study.

### Implications for research and/or practice

Our study provides a scoring system that is very easy to use in daily clinical practice that can facilitate the request for spirometry based on a calculated risk, making it a good model for COPD screening. For its use, once the result has been obtained in smokers who have attended the health care centre (opportunistic screening), the physician must evaluate the need for confirmation by spirometry, according to patient characteristics and cost-effectiveness. In addition, understanding their COPD risk may well encourage smokers who are already considering quitting to take the definitive step^37^. Because of its ease of use, this scoring system can be extended to the entire population through mobile applications (apps), enabling patients to easily understand their risk and thus serving as a coercive measure for quitting. In other words, the points obtained for age cannot be modified, but it is visible on the scale that quitting smoking can improve scores, both in nicotine dependence and in respiratory symptoms. It is also a tool that can save time in primary care consultations and in the use of spirometry by applying the technique only in cases with a high probability of fixed airflow limitation. Finally, this model (and those in Table 1) should be externally validated in other populations and its cost-effectiveness verified in order to be able to extend its use in primary care in other centres and clinical settings. It also raises issues that open lines of investigation in the action against smoking and target population.

## Data Availability

The data that support the findings of this study are available from Esther Román-Conejos but restrictions apply to the availability of these data, which were used under license for the current study, and so are not publicly available. Data are however available from the authors upon reasonable request and with permission of the Clinical Trials Ethics Committee of the Department of Health of the Generalitat Valenciana (General University Hospital of Elche, Alicante).
